# Dr. Wm. A. Hawley

**Published:** 1891-06

**Authors:** 


### OBITUARY.

DR. WILLIAM A. HAWLEY died at his residence, No. 407 Montgomery street, Syracuse, at midnight, May 15th. He had been sick about two months. Dr. Hawley has practiced medicine in Syracuse for about fifty years and attained considerable eminence in his profession.

He was a member of the International Hahnemannian Association and took an active part in the discussions of the last annual meeting, as those of our readers will remember who have perused the reports of the meeting published by this journal from time to time since last June.

